# Development and implementation of a nationwide multidrug-resistant organism tracking and alert system for Veterans Affairs medical centers

**DOI:** 10.1017/ice.2024.79

**Published:** 2024-09

**Authors:** Christopher D. Pfeiffer, Makoto M. Jones, James S. Klutts, Quinn A. Francis, Hannah M. Flegal, Adrienne O. Murray, Tina M. Willson, Natalie R. Hicks, Charlesnika T. Evans, Martin E. Evans

**Affiliations:** 1 Portland VA Health Care System, Portland, OR, USA; 2 University of Oregon, Portland, OR, USA; 3 VA Salt Lake City Health Care System, Salt Lake City, UT, USA; 4 Division of Epidemiology, Department of Internal Medicine, University of Utah School of Medicine, Salt Lake City, UT, USA; 5 National Pathology and Laboratory Medicine Program Office, Veterans Health Administration, US Department of Veterans Affairs, Washington, DC, USA; 6 Iowa City VA Health Care System, Iowa City, IA, USA; 7 University of Iowa Carver College of Medicine, Iowa City, IA, USA; 8 National Infectious Diseases Service, Specialty Care Services, Veterans Health Administration, US Department of Veterans Affairs, Washington, DC, USA; 9 VA Center of Innovation for Complex Chronic Healthcare, Hines VA Hospital, Hines, IL, USA; 10 Department of Preventive Medicine, Center for Health Services and Outcomes Research, Northwestern University, Chicago, IL, USA; 11 Lexington Veterans Affairs Healthcare System, Lexington, KY, USA; 12 Division of Infectious Diseases, Department of Internal Medicine, University of Kentucky School of Medicine, Lexington, KY, USA

## Abstract

**Objective:**

Develop and implement a system in the Veterans Health Administration (VA) to alert local medical center personnel in real time when an acute- or long-term care patient/resident is admitted to their facility with a history of colonization or infection with a multidrug-resistant organism (MDRO) previously identified at any VA facility across the nation.

**Methods:**

An algorithm was developed to extract clinical microbiology and local facility census data from the VA Corporate Data Warehouse initially targeting carbapenem-resistant *Enterobacterales* (CRE) and methicillin-resistant *Staphylococcus aureus* (MRSA). The algorithm was validated with chart review of CRE cases from 2010-2018, trialed and refined in 24 VA healthcare systems over two years, expanded to other MDROs and implemented nationwide on 4/2022 as “VA Bug Alert” (VABA). Use through 8/2023 was assessed.

**Results:**

VABA performed well for CRE with recall of 96.3%, precision of 99.8%, and F1 score of 98.0%. At the 24 trial sites, feedback was recorded for 1,011 admissions with a history of CRE (130), MRSA (814), or both (67). Among Infection Preventionists and MDRO Prevention Coordinators, 338 (33%) reported being previously unaware of the information, and of these, 271 (80%) reported they would not have otherwise known this information. By fourteen months after nationwide implementation, 113/130 (87%) VA healthcare systems had at least one VABA subscriber.

**Conclusions:**

A national system for alerting facilities in real-time of patients admitted with an MDRO history was successfully developed and implemented in VA. Next steps include understanding facilitators and barriers to use and coordination with non-VA facilities nationwide.

## Introduction

Multidrug-resistant organisms (MDROs) are difficult to treat and are associated with high morbidity and mortality and the potential for rapid spread.^1^ Many of these are on the US Centers for Disease Control and Prevention (CDC) lists for “urgent” or “serious” antibiotic-resistant threats.^2^ Prompt identification and isolation of newly admitted patients colonized or infected with MDROs is key to the control of these organisms in healthcare settings.

In 2017, the Veterans Health Administration (VA) developed a nationwide system to track and notify infection prevention and control personnel at local medical facilities in real time when they had an acute care patient or long-term care resident admitted to their facility with a history of being colonized or infected with an MDRO. These admissions included MDROs in Veterans previously hospitalized at their facility or transferred from another facility within the national VA system. While the former was tracked via various methods within a facility, MDROs documented at other (remote) VA facilities were previously unable to be systematically tracked by local Infection Prevention (IP) teams. This tool, which evolved into “VA Bug Alert” (VABA) in 2022 (see Table 1), aimed to address this gap.

**Table 1.** Veterans Health Administration Bug Alert implementation timeline

	2017	2020	2022
Program name	MDRO Tracker	Inpatient Pathogen Tracker	VABA
Pathogens tracked	MRSA, CRE/CP-CRE	MRSA, CRE/CP-CRE	MRSA, CRE/CP-CRE, CRAB, VRE, *C. auris*
Implementation type	Opportunistic via research	Open to all, passive engagement	Active engagement with field

This Table provides the timeline including name of previous versions of VABA, as well as which pathogens were covered and the mode of implementation. Initially, VA facilities were recruited as part of research, and given their ability to take on new programs, it was then open to all medical centers voluntarily, and since 2022, there has been active engagement with all VA health care systems.

This is a report on the development through initial national implementation of VABA, which may serve as a model for other large healthcare networks.

## Methods

### Data sources

**Figure 1.**
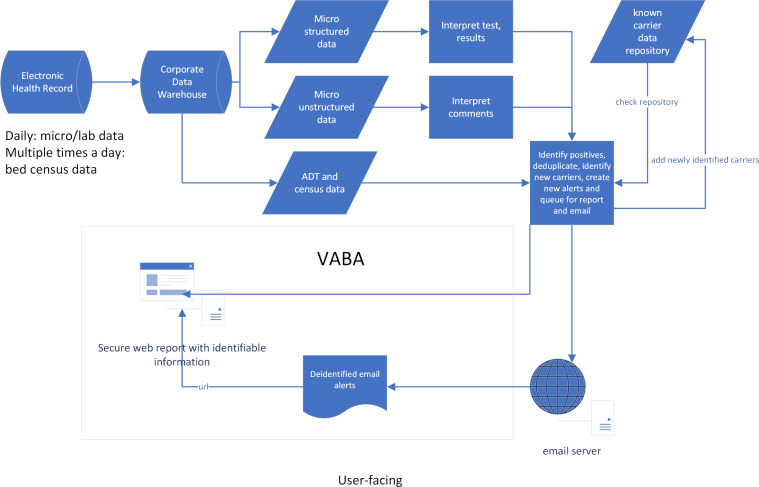
Veterans Health Administration Bug Alert algorithm and Interaction with Veterans Health Administration Data Systems.

Clinical microbiology data for VABA development were obtained from the VA Corporate Data Warehouse (CDW) (see Figure 1). Each night the most current patient/resident care data in the Veterans Health Information Systems and Technology Architecture (VistA) electronic health records at each VA medical facility were downloaded into the CDW. Facility inpatient censuses were updated in near real-time. Veterans infected or colonized with carbapenem-resistant (CRE) and carbapenemase-producing *Enterobacterales* (CP-CRE) or methicillin-resistant *Staphylococcus aureus* (MRSA) were identified in the CDW by employing algorithms to find clinical cultures and molecular tests yielding bacteria with pre-defined phenotypic and/or genotypic antimicrobial resistance.

CRE/CP-CRE were defined in two ways. “CRE” was based on the 2015 CDC CRE guideline^3^ and included *E. coli*, *Klebsiella* spp., and *Enterobacter* spp. that were non-susceptible to imipenem, meropenem, doripenem, or ertapenem, and resistant to any tested 3^rd^ generation cephalosporin. “CP-CRE,” based on the 2017 VA CP-CRE guideline (available upon request), included *E. coli*, *K. pneumoniae, K. oxytoca*, and *Enterobacter* spp. resistant to imipenem, meropenem, or doripenem plus the identification of carbapenemase-producing genes using a nucleic acid amplification test (NAAT). Positive NAAT carbapenemase perirectal/rectal screening tests were included.

MDROs subsequently added to VABA included *Candida auris*, carbapenem-resistant *Acinetobacter baumanii* (CRAB), and vancomycin-resistant *Enterococcus spp.* (VRE). See supplemental material for detailed organism definitions.

All definitions were applied retrospectively. A patient was retained in the database for one year after an MRSA-positive laboratory test and indefinitely for all other pathogens.

### Algorithm development

CRE/CP-CRE and MRSA were selected for initial VABA development. CRE/CP-CRE was targeted due to its emerging global antimicrobial threat status and relatively low incidence within the VA system (approximately 400 reported isolates per year).^2,4^ Due to concerns about too few CRE/CP-CRE cases alone to develop and evaluate VABA, MRSA was selected to provide a more robust dataset due to the experience with and relatively high prevalence of MRSA in the VA system allowing the ability to compare VABA with existing MRSA detection tools.^5,6^

Initially, daily review and an error reporting system of CRE/CP-CRE and MRSA data were used to iteratively improve the sensitivity and specificity of VABA. Laboratory naming conventions for MRSA (described below) have been in place since 2007 and validated thereafter^5^. Based on this precedent, during the trial period, standardized definitions were developed for CRE/CP-CRE and distributed for use by all VA clinical microbiology laboratories. However, notably, individual medical centers to varying degrees engaged in commercial, academic affiliate, in-house, or a combination of these laboratories, resulting in some variations in protocol, platform, and documentation. Because laboratory nomenclature was critical to VABA’s ability to identify CRE/CP-CRE accurately, and because many laboratories reported these organisms using free-text comments, text processing was used to identify and interpret these results in CDW (see Supplement for further details).

### Algorithm validation

To formally assess VABA performance, the CDW was queried for all VA patients/residents with at least one positive CRE/CP-CRE test result during calendar year 2017 and for any additional positive CRE results amongst those patients/residents from January 2010 through December 2018. A detailed chart review of the local microbiology and chemistry laboratory reports of those patients/residents was conducted using VA’s Compensation and Pension Interchange software^7^ to determine if the positive CRE/CP-CRE test recorded in the laboratory package of CDW was supported by data in the patient/resident medical record. These medical records were also reviewed for evidence of additional CRE/CP-CRE results that were not found in the CDW query. Test results were thus categorized as true positives, false positives, or false negative results.

Recall, precision, and the F1 score were used to assess performance of VABA for CRE/CP-CRE because of imbalanced classification.^8^ Recall (the ability of a classification model to identify all relevant instances) was calculated as true positive tests/(true positive tests + false negative tests); precision (the ability of a classification model to return only relevant instances) was calculated as true positive tests /(true positive tests + false positive tests), and the F1 score (a single metric that combines recall and precision using the harmonic mean) was calculated as 2 × ((precision × recall)/(precision + recall)).

All other pathogens underwent informal validation facilitated by program office staff including iterative feedback from the field. *C. auris* and CRAB, due to low prevalence, continued longitudinal validation throughout the project.

### Trial site testing and assessment

The potential VABA utility was assessed by analyzing the subset of admitted patients/residents identified as a probable CRE/CP-CRE or MRSA carrier at 24 participating sites during 2017–2018. For identified patients/residents, the tracking system recorded admission details (admission date and time, facility, ward), MDRO details (specimen date(s), organism, accession number, and specimen type (nares surveillance swab, blood culture, urine culture, etc.). For CRE/CP-CRE and MRSA, the inpatient prevalence, stratified by admission and by patient/resident was analyzed along with the number of automated alerts generated for patients/residents that had been hospitalized with CRE/CP-CRE or MRSA at another (remote) VA facility, ie those patients that would not have otherwise been systematically identifiable by the local facility surveillance mechanisms.

VABA was trialed in the field with 10 sites in 2017 and an additional 14 sites in 2018. Staff participants at each site were recruited based on alignment with job duties related to MDRO prevention and control. Participants were educated on VABA through group webinar training and were provided comprehensive guides. Study investigators at the Portland, Oregon, and Salt Lake City, Utah VAs provided support to individual participants as needed.

**Figure 2.**
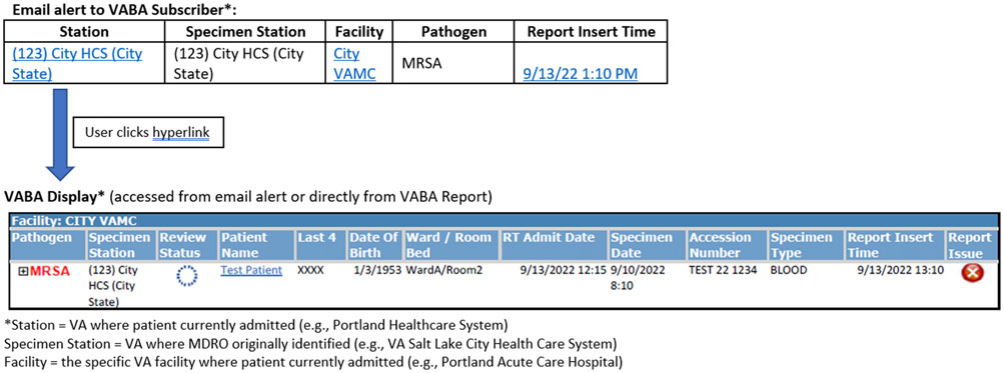
Veterans Health Administration Bug Alert (VABA) User Experience – Email Alert and VABA Display.

Clinical microbiology laboratory data in CDW that met the definitions stated above were used to identify and incorporate Veterans with MDRO colonization or infection into a nationwide registry. This master patient/resident index system was used to subsequently identify admissions in any VA across the US and send an email alert to recruited staff which informed them when a patient/resident colonized or infected with an MDRO was being admitted to their facility (see Figure 2). While the microbiology data refreshed nightly, the admission data refreshed multiple times a day, enabling close to real-time alerts for newly admitted patients with a history of an MDRO. Each email alert provided non-identifiable information (due to privacy regulations) but included the MDRO identified along with a direct link to secure VABA online reports through which participating staff accessed additional information including patient/resident identifiers, admission times, and MDRO details. These additional reports were also available directly from a weblink (email not required) for user convenience such that all admitted or recently discharged patients with history of qualifying MDROs could be reviewed.

User engagement was formally assessed by automatic recordings of the number of times the report was accessed or the alerts subscribed to by all users by type (Infection Preventionist, physician, laboratorian, or other) and facility.

Perceived utility was assessed by an optional feedback link that was made available when users received a VABA notification or queried an individual patient/resident’s data. During the trial phase, users were encouraged to complete the feedback link as frequently as possible and were specifically prompted whenever viewing information about a patient with CRE/CP-CRE. The assessment asked users to evaluate the usefulness of the information provided by selecting one of the following responses:

- I didn’t know this information and probably wouldn’t have.
- I didn’t know this information at the time but would have.
- I was aware of this information, but it was still useful.
- I was aware of this information. The tool was redundant.

In 2021, before national rollout, a survey was administered to VA IPs and MDRO Prevention Coordinators (MPCs) to assess their awareness and knowledge of the tool, ways the tool could be improved, and why the tool was not being used more often or at all at their facility. Survey feedback prompted several changes aimed at improved functionality and utilization including addition of other selected MDROs based on the 2019 CDC AR threat report^2^ including *C. auris*, CRAB, and VRE coupled with user choice of email alerts by MDRO (eg, remote station MRSA plus all *C. auris*); new flag options to mark cases already reviewed to prevent duplication of work; and an added section to free-text case notes to facilitate intra-facility user communication.

The project was reviewed and approved by the VA Portland Institutional Review Board (#4007) and at each participating VA health care facility as applicable.

### Nationwide implementation and early use assessment

VABA was rolled out nationwide in April 2022. Several webinars were given to IPs and MPCs detailing VABA, its use, potential value, and how to subscribe to alerts. Initial VABA use in the field through 8/2023 was measured by counting the number of users overall; number of users by facility including the facility location and complexity level^9^; user roles (IPs/MPCs, charge nurses, MDs, others); the number of alert subscriptions overall and by MDRO type; and the number of times a report was accessed by a unique user.

## Results

### Algorithm validation

Query of the CDW identified 1,982 CRE/CP-CRE-positive laboratory specimens from 2010 through 2018 from 907 unique Veterans identified as having at least one positive CRE result in 2017. Of these, specimens from three unique Veterans were not substantiated (false positive) while the remaining 1,979 specimens were confirmed as CRE or CP-CRE (true positive). Chart review revealed that 75 specimens were missed by the VABA algorithm (false negative). Thus, the calculated recall of VABA was 96.3%; precision was 99.8%, and the F1 score was 98.0%.

### Trial site assessment

There were 277,527 unique Veterans admitted 610,495 times to acute or long-term care units of the 24 trial sites from January 2017 through December 2018. Of these, there were 878 CRE, 461 CP-CRE, and 69,945 MRSA patients with history, admission screen, or clinical culture during the current admission for these organisms giving a prevalence of 0.1, 0.1, and 11.5 cases per 100 admissions for the three MDROs, respectively. Of these, 670 (76%) CRE, 304 (66%) CP-CRE, and 45,524 (65%) MRSA patients could be identified by history alone. Of those admissions identified solely by history, that history was available only from a remote (non-local) facility in 103 (15.4%), 4 (1.3%), and 2,564 (5.6%) of CRE, CP-CRE, and MRSA cases, respectively.

Automatic recordings of the logins and queries of all users showed that IPs and MPCs used VABA most often, followed by Infectious Disease physicians and research staff. As a result, IPs and MPCs provided 91% of the feedback while hospital epidemiologists/infectious diseases physicians, laboratorians, and research personnel provided 7%, <1%, and 2% of the feedback, respectively. Feedback was provided for 1,011 facility admissions with a history of CRE/CP-CRE (130), MRSA (814), or CRE/CP-CRE and MRSA (67). Of these admissions, respondents indicated that they had been previously unaware of the information in 338 (33%) of the cases, and of these, 271 (80%) reported that they would not have otherwise become aware of this information.

### Nationwide early-use assessment

As of August 2023, 113 of VA’s 130 (87%) health care systems had at least one VABA user after full implementation nationwide. These medical centers were located throughout the US and ranged from the least to most complex care levels. Of the 139 persons nationwide subscribed to an alert, 123 subscribed to CRE/CP-CRE alerts, 104 to *C. auris* alerts, 102 to MRSA alerts, 39 to CRAB alerts, and 41 to VRE alerts. The frequency of accessing VABA nationwide averaged 36 unique users per week, with a high of 73 per week.

## Discussion

The Veterans Health Administration is the largest coordinated healthcare system in the United States. Directives for the management of Veterans colonized or infected with MDROs including MRSA and CP-CRE have been developed, revised, and disseminated to all acute and long-term care facilities since 2007, yet there remains a lack of established inter-facility communication when patients or residents are transferred between facilities. In a survey done in 2018, it was found that communication between facilities about Veterans who were colonized or infected with CP-CRE occurred only 38%–42% of the time when the individual was transferred from another VA facility.^10^

Often, infection control interventions designed to prevent spread of MDROs are independently implemented by individual health care facilities without clear coordination with other facilities within a healthcare system that may care for the same patients. Efforts limited to a single facility do not account for the potential of inter-facility spread through movement of patients who are colonized or infected with these organisms. Models representing interconnected health care facilities illustrate that a coordinated approach to interrupting transmission is more effective than independent facility-based efforts. In one study, a coordinated response to prevent CRE across a group of interconnected health care facilities resulted in a cumulative 74% reduction in acquisitions over 5 years in a 10-facility network model and 55% reduction over 15 years in a 102-facility network model.^11^

The aim of this work was to develop, implement, and assess the initial use of a nationwide tracking and alert system for MDROs within the VA system. Accuracy of VABA was initially addressed indirectly through feedback from research staff using the tool at each facility. These data suggested that the tool was well accepted and compared favorably with other surveillance data sources used for identification of MDROs. There was some variability in individual clinical microbiology laboratory reporting, but this was addressed with the use of natural language processing for data mining and efforts to standardize reporting nomenclature.

Although VABA has an excellent recall, precision, and F1 score for CRE/CP-CRE indicating that the program is robust and should be of value in identifying Veterans being transferred between facilities carrying MDROs, its value is dependent upon acceptance and use by individuals at each facility within the VA system. Evolving antimicrobial resistance identification methods, including changes in antimicrobial susceptibility breakpoints, could produce fluctuations in performance and so continuous validation and communication with laboratories is necessary.

As of 8/2023, there was a VABA user in the majority (87%) of VA health care systems nationwide and 36 average users per week. We would like to increase VABA use in all 130 VA medical centers as well as promote higher weekly usage, but there are several possible barriers. First, VABA alone may not be as efficient or adequate as the existing local systems already in place to identify MDROs, and it requires infection prevention staff to learn and incorporate an additional program into their workflow. Second, the system was initially built to target CP-CRE and although additional high-impact organisms have been added, some users may not be sufficiently concerned about CP-CRE given the relative infrequency of CP-CRE in the VA system (eg, 2 CP-CRE healthcare-associated infections [HAIs] reported monthly in 2023) such that VABA adoption may be perceived as lower priority. For example, although a 2017 survey showed that at least 75% of VA facilities nationwide had isolated CRE from at least one patient, there was substantial geographic variability with more isolates being from urban, complex, healthcare facilities and fewer from smaller, rural hospitals.^12^ And in another VA survey, 70% of facilities did not screen admissions for CP-CRE because they did not think they had a problem with these organisms.^10^ Third and finally, because user may select from various email alert options, a potentially engaged user may not receive an alert for months in the event no patients are admitted locally with a qualifying history of the target pathogen (eg *C. auris* or CP-CRE), such that the optimal number of national weekly users is not yet well established.

Another limitation of VABA is that MDROs in Veterans identified with MDROs in community healthcare facilities outside of the VA system are not included in the VA CDW database and would not trigger a VABA alert on admission to a VA facility. Others have shown that communication between facilities about patients carrying MDROs occurred only 15%–22% of the time when the individual was transferred from a non-VA acute or long-term care facility.^11^ This limitation varies by geographic region, and while the VA is a closed healthcare system, this gap in VABA is more pronounced in regions with higher percentage of non-VA care. Implementing VABA within VA without outside data input, however, may still have a strong impact within VA since modeling of a similar program showed benefit when only 25% of facilities participated.^13^ This limitation also may change with adoption of the Veterans Health Administration Veteran Interoperability Pledge wherein VA has agreed to work with 13 large non-VA healthcare systems throughout the US toward “developing a framework to allow VA and community providers to securely exchange information to assist in the care of Veterans receiving treatment inside and outside VA.”^14^

VABA was developed several years after the Illinois eXtensively Drug Resistant Organism (XDRO) registry.^15^ Like VABA, the XDRO registry initially targeted CP-CRE but was then expanded to other MDROs. Currently, the XDRO registry serves as Illinois’ CRE reporting tool (across healthcare systems) and a database that healthcare facilities can query to evaluate whether an admitted patient has a history of an XDRO in Illinois. In contrast, while VABA does not serve as a public reporting mechanism, VABA does not require prospective data submission by individual facilities and is accessible both via query and “push” email notifications to local VA facility personnel in near real-time for MDROs of interest. Each of these systems has its strengths and limitations.

In summary, from 2017–2022 VABA was developed and rolled out nationwide, and as of 2023 most VA medical centers had at least one VABA user. The program fills an important gap in MDRO prevention in the nation’s largest healthcare system. Future work will focus on evaluating and improving user acceptability and engagement, continually improving and adding to the VA database, and developing links to non-VA facilities.

## Supporting information

Pfeiffer et al. supplementary material Pfeiffer et al. supplementary material

## Supplementary material

The supplementary material for this article can be found at https://doi.org/10.1017/ice.2024.79
