# Supplementary material for: Development and implementation of a nationwide multidrug-resistant organism tracking and alert system for Veterans Affairs medical centers
Source: Infect Control Hosp Epidemiol. 2024 May 24;45(9):1073–8. doi: 10.1017/ice.2024.79 (PMC11518666; doi:10.1017/ice.2024.79)
Supplement: Pfeiffer et al. supplementary material [file S0899823X24000795sup001.docx]

**VABA Supplement:**

1. **VABA Pathogen Definitions**

| **Pathogen** | **Description** |
| --- | --- |
| Methicillin-resistant *Staphylococcus aureus* (MRSA) | Any positive methicillin-resistant *staphylococcus aureus* result within 365 days. |
| Carbapenem-resistant *Enterobacterales* (CRE) | Any positive *E. coli, Klebsiella spp., and Enterobacter spp.* testing non-susceptible to imipenem, meropenem, doripenem, or ertapenem, and resistant to any tested 3^rd^ generation cephalosporin. Based on 2015 CDC CRE guideline. |
| Carbapenemase-producing carbapenem-resistant *Enterobacterales* (CP-CRE) | Any positive *E. coli, K. pneumoniae, K. oxytoca, and Enterobacter spp.* with resistance to imipenem, meropenem, or doripenem and identification of carbapenemase-producing genes using a nucleic acid amplification test (NAAT); or any positive peri-rectal NAAT screening test. Based on 2017 VA CP-CRE guidelines. |
| *Candida auris* | Any positive *Candida auris* culture or lab chemistry result. |
| Vancomycin-resistant *Enterococcus spp* (VRE) | Any positive, glycopeptide-resistant *Enterococcus spp* culture. |
| Carbapenem-resistant *Acinetobacter baumannii* (CRAB) | Any positive *Acinetobacter* *baumannii* culture with non-susceptibility to imipenem, meropenem, or doripenem or identification of carbapenemase-producing genes. |

| 1. **VABA CRE CPE comment processing**   1. All comments are concatenated (i.e., Specimen comment, Antibiotic sensitivity comment, gram stain, bacteriology preliminary comment, bacteriology report remark, bacteriology smear prep, and microbiology comments are concatenated) |
| --- |
| 2. All phrases are identified within comments with start and stop position noted |
| 3. Phrases classified as one of (subjectkey, positiveverb, negativeverb, negativekey, negator) |
| 4. When negative phrases are proximally closer to the predicate then negate. When positive phrases are closer then confirm. |
| 5. When a carbapenemase phrase is present and not negated then count as confirmed. |
| 6. integrate with structured data. |

1. **VABA CRE CPE Text Processing**

| CKId | Category | KeyPhrase |
| --- | --- | --- |
| 1 | SubjectKey | KPC |
| 2 | SubjectKey | Carbapenemase |
| 3 | SubjectKey | CARBAPENEMASE-PRODUCING ENTEROBACTERIACEAE |
| 4 | SubjectKey | CP-CRE |
| 5 | SubjectKey | KLEBSIELLA PNEUMONIAE + CARBAPENEMASE |
| 6 | SubjectKey | Carb-R |
| 7 | SubjectKey | Carba NP |
| 8 | NotUse | CARBAPENEM |
| 9 | NotUse | CARBAPENEM RESISTANT ENTEROBACTERIACEAE |
| 10 | NotUse | CRE |
| 11 | NotUse | CARBAPENEM RESISTANT KLEBSIELLA PNEUMONIAE |
| 12 | NotUse | CARBAPENEMS |
| 13 | SubjectKey | CARBAPENEMASE RESISTANT PSEUDOMONAS AERUGINOSA |
| 14 | SubjectKey | IMP-1 |
| 15 | SubjectKey | VIM |
| 16 | SubjectKey | NDM |
| 17 | SubjectKey | OXA-48 |
| 18 | SubjectKey | Carbepenem-Resistant Enterobacteriae |
| 19 | SubjectKey | KLEBSIELLA PNEUMONIAE CARBAPENEMASE |
| 20 | SubjectKey | Enterobcteriaceae Carbapenemase |
| 21 | NotUse | ESBL (+) CARBAPENEMASE |
| 22 | SubjectKey | ESBL + CARBAPENEMASE |
| 23 | NotUse | ESBL |
| 24 | SubjectKey | ESBL+CARBAPENEMASE |
| 25 | NotUse | Extended Spectrum Beta Lactamase |
| 26 | SubjectKey | HODGE TEST |
| 27 | NotUse | IMIPENEM |
| 28 | NotUse | Klebsiella pneumoniae |
| 29 | SubjectKey | OXA |
| 30 | SubjectKey | NDM-1 |
| 31 | SubjectKey | New Delhi metallo-beta-lactamase |
| 32 | SubjectKey | OXA48 |
| 33 | SubjectKey | blaKPC |
| 34 | NotUse | KLEB PNEUMO |
| 35 | PositiveVerb | Detected |
| 36 | PositiveVerb | Positive |
| 37 | PositiveVerb | Confirmed |
| 38 | PositiveVerb | Present |
| 39 | PositiveVerb | Identified |
| 40 | PositiveVerb | Recovered |
| 41 | PositiveVerb | POS |
| 42 | NotUse | KNOWN |
| 43 | NegativeVerb | Not Detected |
| 44 | NegativeVerb | Negative |
| 45 | NegativeVerb | Not Confirmed |
| 46 | NegativeVerb | Not indicated |
| 47 | NegativeVerb | Absent |
| 48 | NegativeVerb | NEG |
| 49 | NegativeVerb | IS Not |
| 51 | Negator | Not |
| 52 | Negator | No |
| 53 | SubjectKey | IMP |
| 54 | NegativeVerb | Not Detect |
| 55 | NotUse | NO INTERPRETATION |
| 56 | SubjectKey | ISOLATE DEMONSTRATES CARBAPENEMASE PRODUCTION |
| 57 | NotUse | NO ZONE |
| 58 | NotUse | NOT BEEN VERIFIED |
| 59 | NotUse | NOT NECESSARILY DEMONSTRATE |
| 60 | NotUse | NO CLSI |
| 61 | Negator | Non |
| 62 | NotUse | not been established |
| 63 | NegativeVerb | NOT DECTECTED |
| 65 | NotUse | not available |
| 66 | NotUse | NO INTERPRETIVE |
| 67 | NotUse | NO IN |
| 68 | NotUse | NOT AN ESBL PRODUCER |
| 69 | NotUse | NOT OFFE |
| 71 | NotUse | not been cleared |
| 72 | NegativeVerb | Carba NP Test Negative |
| 73 | NegativeVerb | not a carbapenemase |
| 75 | SubjectKey | CRE-KPC |
| 76 | SubjectKey | KPC-CRE |
| 77 | SubjectKey | CARBEPENEMASE |
| 78 | SubjectKey | KPC+ |
| 79 | NotUse | CARBAPENEM-RESISTANT ENTEROBACTERIACEAE |
| 80 | NotUse | IMIPENEM RESISTANT |
| 81 | NotUse | GRAM NEGATIVE |
| 82 | NotUse | OXIDASE NEGATIVE |
| 83 | SubjectKey | Carbapenem-Resistance Co |
| 84 | NotUse | NOT RELIABLE |
| 85 | SubjectKey | Carbapenemase-resistant Enterobacteriaceae |
| 86 | NotUse | GRAM POSITIVE |
| 87 | SubjectKey | blaNDM |
| 88 | SubjectKey | blaVIM |
| 89 | SubjectKey | blaOXA |
| 90 | SubjectKey | blaIMP |
| 91 | NegativeVerb | no evidence of Carbapenemase |
| 92 | NegativeVerb | confirmed HODGE TEST IS NEGATIVE |
| 93 | NotUse | non-susceptibility |
| 94 | NotUse | non - susceptibility |
| 95 | NegativeVerb | ISOLATION NOT REQUIRED |
| 96 | NegativeVerb | No genetic determinants for carbapenem non-susceptibility detected |
| 97 | NegativeVerb | No carbapenemase resistance genes detected |
| 98 | NegativeKey | No Carbapenemase detected |
| 99 | NotUse | not intended to guide or monitor treatment of infection |
| 100 | NotUse | negative result does not exclude the presence |
| 101 | NotUse | negative result does not exclude the presence |
| 102 | PositiveVerb | RESISTANCE DUE TOd |
| 103 | NotUse | No SpecimenComment |
| 104 | NotUse | No BacteriologyPreliminaryComment |
| 105 | NotUse | No GramStain |
| 106 | NotUse | No AntibioticSensitivityComments |
| 107 | NotUse | No BacteriologyReportRemark |
| 108 | NegativeVerb | NO CARB-R GENES DETECTED |
| 109 | NotUse | not included |
| 110 | NotUse | No BacteriologyPreliminaryComme |
| 111 | NotUse | No BacteriologyPrelimin |
| 112 | NotUse | No Bacteri |
| 113 | NotUse | not recorded |
| 114 | NotUse | NEUTROS NO ORGANISMS SEEN |
| 115 | NegativeVerb | NOT been confirmed |
| 116 | NotUse | No BacteriologySmearPrep |
| 117 | NotUse | No WBC |
| 118 | NotUse | No growth |
| 122 | NotUse | PREVIOUS POSITIVE CRE |
| 123 | NotUse | WBC Present |
| 124 | NegativeVerb | UNDETECTED |
| 125 | NegativeVerb | UNDETECT |
| 126 | NegativeVerb | ND |
| 127 | NotUse | Possible carbapenem resistant Enterobacteriaceae ( CRE ) present |
| 128 | NotUse | gram negative rods |
| 129 | NotUse | Confirmed ESBL positive isolate |
| 130 | NotUse | Nitrofurantoin is not recommended |
| 131 | NotUse | Cefazolin may not predict Cephalexin susceptibility |
| 134 | SubjectKey | Carbapenemase Producing- Carbapenem Resistant Enterobacteriaceae |
| 135 | NotUse | Carbapenem Resistant Enterobactericeae ( CRE ) isolated |
| 136 | NotUse | Presumptive CP-CRE ( Carbapenemase Producing-Carbapenem Resistant Enterobacteriaceae |
| 137 | NotUse | Critical value for CRE called to and read back |
| 138 | NotUse | MDRO CRE NOTIFIED |
| 139 | NotUse | CRE TEST Not Performed |
| 140 | NotUse | PRESUMPTIVE CP-CRE |
| 141 | NotUse | GRAM NEGATIVE |
| 142 | NotUse | NO POLYMORPHONUCLEAR |
| 143 | NotUse | NO ORGANISMS |
| 144 | NotUse | Negative for Extended Spectrum Beta Lactamase |
| 145 | NotUse | no published Fosfomycin |
|  |  |  |
| 147 | NotUse | WARD PREVIOUSLY NOTIFIED OF POSITIVE RESULTS |
|  |  |  |
|  |  |  |
| 150 | NotUse | GRAM NEGATIVE ROD OXIDASE NEGATIVE |
| 151 | NotUse | CULTURE NEGATIVE TO DATE |
| 152 | SubjectKey | Modified Hodge Test-Positive |
| 153 | NotUse | NO ANAEROBES ISOLATED |
| 154 | NotUse | may not respond to therapy |
| 155 | NotUse | EXTENDED SPECTRUM BETA-LACTAMASE DETECTED |
| 156 | NotUse | PROBABLY NOT Carbapenemase producing Klebsiella pneumoniae |
| 157 | SubjectKey | ESBL + CARBAPENEMASE POSITIVE |
| 158 | NotUse | Negative for PBP2a |
| 159 | NotUse | Susceptibility not performed |
| 160 | NotUse | POSSIBLE KPC PRODUCER |
| 161 | NotUse | ISOLATE MAY NOT NECESSARILY DEMONSTRATE CARBAPENEMASE |
| 162 | NotUse | GRAM STAIN Not Performed |
| 163 | NotUse | AZTREONAM SHOULD NOT BE USED |
| 164 | NotUse | NO EPITHELIAL CELLS SMEAR |
| 165 | NotUse | NO EPITHELIAL CELLS |
| 166 | NotUse | CARBA-R PANEL Not Performed |
| 167 | NotUse | NO FURTHER WORKUP |
|  |  |  |
| 169 | NotUse | NO ORAL FLORA TYPES |
| 170 | NotUse | NO OBLIGATE ANAEROBES |
| 171 | NotUse | GRAM NEG ORGANISM |
| 172 | NotUse | POSITIVE CULTURE RESULTS READ BACK |
| 173 | NotUse | ANAEROBES ARE NOT DONE |
| 174 | NotUse | HISTORY OF CRE |
| 175 | NotUse | BacteriologyReportRemark : NO GROWTH |
| 176 | NotUse | HAVE NOT PROVEN EFFECTIVE |
| 177 | NotUse | TESTING IS NOT A RELIABLE |
| 178 | SubjectKey | METALLO-CARBAPENEMASE |
| 179 | SubjectKey | CARBAPENAMASE PRODUCER |
| 180 | SubjectKey | KPC PRODUCER |
| 181 | NotUse | It has not been cleared |
| 182 | NotUse | approval is not necessary |
| 183 | SubjectKey | CRE-CONFIRMED |
| 184 | SubjectKey | CARBAPENAMASE PRODUCER-HODGE TEST |
| 185 | NotUse | NO ESTAB. NORMALS |
| 186 | NotUse | no LSI std.s |
| 187 | NotUse | NO PHONE CALLS |
| 188 | SubjectKey | KPC-CONFIRMED |
| 189 | NotUse | BETA-LACTAM DRUG IS NOT RELIABLE |
| 190 | NotUse | LABORATORY DOES NOT PROVIDE TESTING |
| 191 | NotUse | TEST SHOULD NOT BE USED FOR DIAGNOSIS |
| 192 | NotUse | IS NOT RELIABLE |
| 193 | NotUse | NO LEGIONELLA |
| 194 | NotUse | NO ACTINOMYCES |
| 195 | NotUse | ESBL - NEGATIVE |
| 196 | NotUse | ESBL NEGATIVE |
| 197 | NotUse | NO ESTAB. |
| 198 | NotUse | (no LSI std.s |
| 199 | NotUse | no LSI std |
| 200 | NotUse | no ESTAB |
| 201 | SubjectKey | KPC-AMPC |
| 202 | NotUse | Modified Hodge Test not performed |
| 203 | NotUse | not for diagnostic purposes |
| 204 | NotUse | NOT BEEN ESTEBLISHED |
| 205 | NotUse | ESBL=NEG |
| 206 | NotUse | ESBL = NEGATIVE |
| 207 | NotUse | ESBL = NEG |
| 208 | NotUse | NOT HAVE ESTABLISHED |
| 209 | NotUse | PHARYNGEAL FLORA ABSENT |
| 210 | SubjectKey | CARBAPENAMSE |
| 211 | NotUse | POSITIVE CRE SCREEN THIS IS JUST A SCREEN AND NOT |
| 212 | NotUse | ESBL POSITIVE |
| 213 | NotUse | Recovered after 1 day of incubation |
| 216 | NotUse | Carbapenem resistant Enterobacteriaceae ( CRE ) present . Further testing in progress . |
| 217 | NotUse | Carbapenem resistant Enterobacteriaceae ( CRE ) present . Further testing in progress . |
| 218 | NotUse | Carbapenem resistant Enterobacteriaceae ( CRE ) present . Further testing in progress . |
| 220 | NotUse | CONFIRMED BY MODIFIED HODGE TEST Report sent to MRSA Coordinator |
| 222 | NotUse | Testing in progress CARBAPENEMASE TESTING IN PROGRESS |
| 223 | NotUse | PRESUMPTIVE Carbapenemase-Resistant Enterobacteriaceae |
| 224 | NotUse | POSSIBLE CRE |
| 225 | NotUse | AmpC Positive |
|  |  |  |
|  |  |  |
| 228 | NotUse | MICRO on presumtive CRE |
| 229 | NotUse | isolates with carbapenem zone diameters |
| 230 | NotUse | NO INTERP Test Performed |
| 231 | NotUse | NO ORGANISMS SEEN |
| 232 | NotUse | NO normal flora present |
| 233 | NotUse | Imipenem reported as S |
| 234 | NotUse | IMIPENEM IS DRUG OF CHOICE |
| 235 | NotUse | MRSA , VRE , ESBL , CRE , C . difficile |
| 236 | NotUse | HISTORY OF BOTH KPC AND |
| 237 | NotUse | NOT PERFORMED |
| 238 | NotUse | NO PATHOGENIC NEISSERIA ISOLATED |
| 239 | NotUse | NO WBCs |
|  |  |  |
| 241 | NotUse | report not final |
| 242 | NotUse | POSSIBLE ESBL AND CARAPENEMASE ( METALLO- OR KPC ) RESISTANT CARBAPENEMS |
| 243 | NotUse | not due to ESBLs |
| 244 | NotUse | NO ORGANISM SEEN |
| 245 | NotUse | CONFIRMED EXTENDED SPECTRUM BETA LACTAMASE |
| 246 | NotUse | CRE = CARBAPENEM RESISTANT ENTEROBACTER |
| 247 | NotUse | KLEBSIELLA PNEUMONIAE SPP PNEUMONIAE PRESUMPTIVE CARBAPENEMASE |
| 250 | NotUse | KPC / NDM testing was performed |
| 251 | NotUse | KNOWN CRE |
| 252 | NegativeKey | IMIPENEM IS SENSITIVE |
| 254 | NotUse | this set is positive |
| 255 | NotUse | ISOLATES HARBORING CARBAPENEMASE |
| 256 | NotUse | KLEBSIELLA PNEUMONIAE NO FURTHER WORKUP |
| 257 | NotUse | positive UTI Screen |
| 258 | NotUse | COAGULASE POSITIVE |
| 259 | NotUse | ML KLEBSIELLA PNEUMONIAE AND |
| 260 | NotUse | POSSIBLE KPC |
| 261 | NotUse | POSSIBLE KPC ( RESISTANT CARBAPENEMS ) |
| 262 | NotUse | INTESTINAL FLORA PRESENT |
| 263 | NotUse | Known history |
| 264 | NotUse | POSSIBLE CARPAPENAMASE RESISTANT KLEBSIELLA PNEUMONIAE |
| 265 | NotUse | PREVIOUSLY TESTED FOR KPC PRODUCTION |
| 266 | NotUse | PREVIOUSLY TESTED POSITIVE FOR KPC |
| 267 | NotUse | Probable Carbapenemase producing |
|  |  |  |
| 269 | NotUse | ESBL IS NON DETERMINATE |
| 270 | NotUse | PREVIOUS RESULT IMP REPORTED |
| 271 | NotUse | PENDING KPC |
| 272 | NotUse | KPC AND Extended Spectrum Beta Lactamase confirmatory test PENDING |
| 273 | NotUse | ANAEROBIC BOTTLE POSITIVE |
| 274 | NotUse | Aerobic positive |
| 275 | NotUse | NEGATIVE = E . S . B . L |
| 276 | NotUse | KPC = AMP C + PORIN LOSS |
| 277 | NotUse | NO BACTERIA SEEN |
| 280 | NotUse | CARBAPENEM RESISTANT TYPE KLEBSIELLA PNEUMONIAE ( KPC ) Test performed |
| 281 | NotUse | CARBAPENEMASE / ESBL TESTING PENDING |
| 282 | NotUse | CARBAPENEMASE / ESBL TESTING PENDING CARBAPENEM RESISTANT TYPE KLEBSIELLA ( KPC ) |
| 283 | NotUse | PENDING KPC confirmatory test |
| 284 | NotUse | E S B L : POSITIVE |
| 285 | NotUse | POSITIVE GRAM NEGATIVE RODS |
| 286 | NegativeKey | RULE OUT KPC |
| 287 | NotUse | PENDING KPC |
| 288 | NotUse | PENDING KPC confirmatory test |
| 289 | SubjectKey | K P C |
| 290 | NotUse | GRAM NEG RODS |
| 291 | NotUse | MODIFIED HODGE TEST WILL BE PERFORMED |
| 293 | NotUse | KPC / NDM-1 PCR TESTING WAS DEVELOPED |
| 294 | NotUse | NO SET BREAKPOINTS |
| 295 | NotUse | COAGULASE NEGATIVE STAPH |
| 296 | NotUse | KPC / NDM-1 PCR assays were performed |
| 297 | NotUse | KPC / NDM-1 PCR assays performed |
| 298 | NotUse | CONFIRMED ESBL |
| 299 | NegativeKey | KPC . RULED OUT |
|  |  |  |
|  |  |  |
| 302 | NotUse | ESBL Confirmed |
| 303 | NotUse | PRESUMPTIVE CRE |
| 304 | NotUse | CP-CRE NOT COMFIRMED |
| 305 | NotUse | PREVIOUSLY CONFIRMED CRE-KPC GENE POSITIVE |
| 306 | NegativeKey | KNOWN CP-CRE RULED OUT |
| 307 | NotUse | CARBAPENEMASE PCR PERFORMED |
|  |  |  |
| 309 | NotUse | KPC NOTIFICATION DELAYED |
| 310 | SubjectKey | NDM-1 PCR |
| 311 | SubjectKey | KPC PCR |
| 312 | SubjectKey | KPC PCR : |
| 313 | SubjectKey | NDM-1 PCR : |
| 314 | NotUse | FOSFOMYCIN NOT ACTUALLY TESTED |
| 315 | NotUse | OXA , IMP , and VIM PCR TESTING PEFORMED |
| 317 | NotUse | PROBABLE KLEBSIELLA PNEUMONIAE CARBAPENEMASE ( METALLO- OR KPC ) |
| 318 | NotUse | PROBABLE CARBAPENEMASE ( METALLO-OR KPC ) |
| 319 | NotUse | PROBABLE CARBAPENEMASE ( METALLO- OR KPC ) |
| 320 | NotUse | POSITIVE FOR CARBAPENEMASE PREVIOUSLY KNOWN |
| 321 | NotUse | PRESUMPTIVE KPC |
| 322 | NotUse | PRIOR + KPC |
| 323 | NotUse | POSSIBLE CARBAPENEMASE RESISTANT |
|  |  |  |
| 325 | NotUse | POSSIBLE CARBAPENEMASE ( METALLO-OR KPC ) |
| 328 | NotUse | PRIOR POSITIVE ESBL , KPC , AMPC |
| 329 | NotUse | PRIOR POSITIVE ESBL / KPC |
| 330 | NotUse | Carbapenemase confirmation performed |
| 331 | NotUse | POSSIBLE KPC CARBAPENEMASE |
| 332 | NotUse | PREVIOUSLY CALLED FOR E S B L AND CARBAPENEMASE RESISTANT |
| 333 | NotUse | POSSIBLE KPC CARBAPENEMASE |
| 334 | SubjectKey | HODGE RESULTS |
| 335 | NotUse | FOR HODGE TEST CONFIMATION |
| 336 | NotUse | HODGE TEST = INDETERMINATED |
| 337 | NotUse | STATE LAB FOR HODGE TEST |
| 338 | NotUse | HODGE TEST PREVIOUSLY POSITIVE |
| 339 | NotUse | SEE # 605 FOR HODGE TEST CONFIRMATION |
|  |  |  |
| 341 | NotUse | PREVIOUSLY MHT POSITIVE |
|  |  |  |
| 344 | NotUse | PREVIOUS POSITIVE MODIFIED HODGE TEST |
|  |  |  |
|  |  |  |
| 347 | NotUse | PREVIOUS KPC |
| 348 | NotUse | HODGE TESTING FOR CARBAPENEMASE RESISTANCE PREVIOUSLY PERFORMED |
| 350 | NotUse | KPC OR CRE ( CARBAPENEM RESISTANT ENTEROBACTERIACEAE ) PRIOR CONFIRMED |
| 351 | NotUse | PREVIOUS POSITIVE FOR KPC |
| 352 | NotUse | Blood Culture POSITIVE |
| 353 | NotUse | Anaerobic Bottle = POSITIVE |
| 354 | NotUse | PRIOR HISTORY OF ESBL , AMPC & KPC |
| 355 | NotUse | PROBABLE CARBAPENEMASE RESISTANT |
| 356 | NotUse | Multi-Drug Resistant Organism Detected |
| 357 | NotUse | Leukoesterase strip positive |
| 358 | NotUse | Nitrite strip negative |
|  |  |  |
| 360 | NotUse | PRESUMPTIVE CARBAPENEMASE-PRODUCING |
| 361 | NotUse | HISTORY OF THIS MDRO KPC |
| 362 | NotUse | PREVIOUS KPC |
| 363 | NotUse | PREVIOUS HODGE TEST |
| 364 | NotUse | Probable KPC |
| 365 | NotUse | GRAM NEG ROD |
| 366 | NotUse | FOR MODIFIED HODGE TEST AND COLISTIN |
| 367 | NotUse | PREVIOUSLY POSITIVE FOR MODIFIED HODGE TEST |
| 368 | NotUse | PROBABLE CARBAPENEMASE |
| 370 | NotUse | DISREGARD RESULT BELOW * * CARBAPENEMASE |
| 371 | NotUse | KPC TESTING UNDERWAY |
| 372 | NotUse | PREVIOUSLY IDENTIFIED AS CARBAPENEMASE |
| 373 | NotUse | no site specified |
| 374 | NotUse | No collection |
| 375 | NotUse | E . S . B . L . NEGATIVE |
| 376 | NotUse | CARBAPENEMASE PRODUCER REFER TO PREVIOUS CULTURES |
| 377 | NotUse | NEISSERIA ABSENT |
| 378 | NotUse | Pharyngeal Flora Absent |
| 379 | NotUse | RESPIRATORY FLORA ABSENT |
| 381 | SubjectKey | MHT |
|  |  |  |
|  |  |  |
| 384 | NotUse | KPC PENDING |
| 385 | NegativeKey | CP-CRE WAS RULED OUT |
|  |  |  |
|  |  |  |
|  |  |  |
| 389 | SubjectKey | KPC-CRE : |
| 390 | SubjectKey | IMP-CRE : |
| 391 | SubjectKey | VIM-CRE : |
| 392 | SubjectKey | NDP-CRE : |
| 393 | SubjectKey | OXA48-CRE : |
| 394 | PositiveVerb | =DETECTED |
| 395 | SubjectKey | Confirmed Carbapenem Producing |
| 396 | NegativeKey | CARBAPENAMASE PRODUCTION NEGATIVE TESTED |
| 397 | SubjectKey | Carbapenemase-producing Carbapenem resistant Enterobacteriaceae |
| 398 | SubjectKey | Carbapenemase-producing Carbapenem resistant |
| 399 | NotUse | KLEBSIELLA PNEUMONIAE CARBAPENEMASE PRODUCER REFER TO PREVIOUS CULTURES |
| 400 | NegativeKey | CARBAPENEM RESISTANT ENTEROBACTERIACEAE CONFIMATION RESULTS : NOT DETECTED |
| 401 | NotUse | EXTENDED SPECTRUM BETA-LACTAMASE POSITIVE |
| 402 | SubjectKey | GENE-KPC |
| 403 | NegativeKey | NO CARBAPENEM RESISTANCE MECHANISM DETECTED |
| 404 | NotUse | Presumptive Carbapenemase Producing-CRE |
| 405 | NegativeKey | No carbapenem resistence genes ( KPC , VIM , IMP , OXA-48 , NDM ) detected |
| 406 | NegativeKey | NOT CRE |
